# Supplementary material for: Rikkosan’s Short-Term Analgesic Effect on Burning Mouth Syndrome: A Single-Arm Cohort Study
Source: Biomedicines. 2024 May 4;12(5):1013. doi: 10.3390/biomedicines12051013 (PMC11118399; doi:10.3390/biomedicines12051013)
Supplement: Supplementary file 1 [file biomedicines-12-01013-s001.zip › TableS1.pdf]

Table S1. Pain scores and treatment periods.

ID	Pre	Post	Treatment periods	The duration of pain complaint	Under treatment with other medications
1	5	5	32	<2 years	Yes
2	6	5	22	<10 years	Yes
3	5	3	31	<10 years	Yes
4	5	2	19	4–5 years	Yes
5	6	2	19	1–2 years	Yes
6	10	3	36	4 years	Yes
7	4	4	35	1 years	Yes
8	5	4	43	5 months	Yes
9	7	3	32	5 months	Yes
10	7	3	34	1 year	Yes
11	10	8	33	2 years	Yes
12	9	8	20	1 year	Yes
13	2.5	1	35	1 year	Yes
14	4.5	1.5	21	4 months	Yes
15	3	2.5	29	4 months	Yes
16	3	3	28	6 months	Yes
17	6	4.5	28	3 months	No
18	7	6.5	30	1.5 years	Yes
19	10	6	35	1.5 years	Yes
20	2	0	28	3 months	No
